# Relationship Between Pregnancy and Acute Disseminated Encephalomyelitis: A Single-Case Study

**DOI:** 10.3389/fimmu.2020.609476

**Published:** 2021-02-01

**Authors:** Shuwen Deng, Ke Qiu, Ranran Tu, Haiping Zheng, Wei Lu

**Affiliations:** ^1^ Department of Neurology, The Second Xiangya Hospital, Central South University, Changsha, China; ^2^ Department of Neurology, The Third Hospital of Changsha, Changsha, China

**Keywords:** acute disseminated encephalomyelitis, pregnancy, immunological pathogenesis, T-helper lymphocytes, Immune Tolerance

## Abstract

The relationship between pregnancy and autoimmune diseases is unclear. This study investigated the possible role of local immune changes and the activation state of the HMGB1/TLR4/Nf-κB/IL-6 pathway at the maternal–fetal interface during pregnancy in the pathogenesis of acute disseminated encephalomyelitis (ADEM). Clinical data and blood samples of a patient with ADEM were collected to observe the dynamic changes in lymphocyte populations after an abortion. The expression of HMGB1, TLR4, Nf-κB, AQP4, IL-2, IL-4, IL-6, and TNF-α in the fetal membrane and placenta was compared between the patient with pregnancy-related ADEM and a woman with a normal pregnancy using Real-time qPCR and western blotting (WB). The patient was diagnosed with ADEM in the early stage of pregnancy after showing limb weakness symptoms. In the third month of gestation, the symptoms worsened, with a disturbance of consciousness and breathing. After the abortion, the patient relapsed with vertigo and visual rotation. Analysis of lymphocyte subsets by flow cytometry showed that B lymphocytes increased, while natural killer T lymphocytes decreased. WB and Real-time qPCR showed that the expression levels of HMGB1, TLR4, Nf-κB, AQP4, and IL-6 in the fetal membrane and placenta were higher in the patient with pregnancy-related ADEM than in the woman with a normal pregnancy, while those of IL-2 were lower in the patient than in the woman with a normal pregnancy. The local immune changes and the activation of the HMGB1/TLR4/Nf-κB/IL-6 pathway at the maternal–fetal interface may be related to the pathogenesis of ADEM.

## Introduction

Acute disseminated encephalomyelitis (ADEM), a postinfectious autoimmune-mediated inflammatory disorder of the central nervous system (CNS), is characterized by widespread demyelination, predominantly involving the white matter of the brain and spinal cord (1). ADEM mainly occurs in children and is usually caused by a viral infection or vaccination. The triggers for immune responses in ADEM are unknown, but the two most widely accepted hypotheses include molecular mimicry and self-sensitization, secondary to CNS infection (2). These two hypotheses involve complex immune responses, which implicate both T-helper type 1 (Th1) and type 2 (Th2) cytokines, with autoreactive T cells playing a key role in ADEM progression (3–5). Th1 cells produce cytokines such as interferon-γ, interleukin (IL)-2, tumor necrosis factor (TNF)-α, and lymphotoxin, which are commonly associated with cell-mediated immune responses against intracellular pathogens, delayed-type hypersensitivity reactions, and induction of organ-specific autoimmune diseases (6). Meanwhile, Th2 cytokines produce anti-inflammatory cytokines, such as IL-4, IL-5, IL-6, and IL-10, which are associated with an enhanced humoral response (7). It is currently believed that the pathogenesis of ADEM is related to the Th2 cytokine-mediated enhancement of humoral immunity (4).

Only a few cases of pregnancy-associated ADEM have been reported, and the underlying mechanism is still unclear (8–11). Under healthy immune conditions, Th1 and Th2 cytokines are in a state of dynamic balance, thus maintaining normal cellular and humoral immune responses (12, 13). However, during pregnancy, immune tolerance is established to promote the growth and development of the embryo (14). Complex immune alterations occur both locally, at the maternal–fetal interface, and systemically. The most obvious changes in the systemic circulation are a shift from a Th1 toward a Th2 immune response and increased activation of innate immune cells (15). The maternal–fetal interface, which is composed of placental trophoblastic and maternal decidual cells (16), also regulates the Th1/Th2 ratio by producing specific cytokines and secreting hormones, such as estrogen, chorionic gonadotropin, and glucocorticoids (17). In addition, immunological alterations may occur during the course of pregnancy, owing to a dynamic shift in the balance of proinflammatory and anti-inflammatory responses (17) and in the concentrations of sex steroids, including estradiol, estriol, and progesterone (18). As ADEM is a Th2 cytokine-mediated autoimmune disease, an enhanced Th2-cell function during pregnancy, at the maternal–fetal interface or systemically, may induce or aggravate the pathogenesis of ADEM in some individuals.

After viral infection or vaccination, the signaling pathway of high mobility group B1 (HMGB1)/Toll-like receptor 4 (TLR4)/Nf-κB is activated (19, 20) and can further induce specific immune responses, promote T-cell differentiation, and induce the activation and release of cytokines such as IL-6 and IL-1 (21, 22). The expression level of IL-6, as a Th2 cytokine, may be closely related to the occurrence of ADEM (23–25). In addition, activation of the HMGB1/TLR4/Nf-κB pathway can regulate the expression level of aquaporin 4 (AQP4) (26). Previous studies have found that AQP4 is associated with CNS inflammatory demyelinating diseases, such as neuromyelitis optical spectrum disorder (NMOSD) (27). It is believed that the pathological mechanism of ADEM is similar to that of NMOSD and is related to the activation of astrocytes (28). Therefore, changes in the expression of AQP4 may also be involved in the pathogenesis of ADEM (29).

During pregnancy, the expression level of Th2 cytokines is elevated at the maternal–fetal interface, and immune tolerance is gradually established (7, 30). Thus, pregnancy may act as an ADEM inducer. Additionally, activation of the HMGB1/TLR4/Nf-κB signaling pathway may influence the expression of IL-6 and AQP4 and, consequently, affect the development of ADEM. Furthermore, AQP4 is an important protein in the fluid exchange between the mother and the fetus (31, 32), and changes in AQP4 expression at the maternal–fetal interface during pregnancy may affect the pathogenesis and recurrence of ADEM.

In this study, we investigated the effects of changes at the maternal–fetal interface during pregnancy on the pathogenesis and recurrence of ADEM, as well as the possible role of the HMGB1/TLR4/Nf-κB/IL-6 pathway in the pathogenesis of pregnancy-related ADEM.

## Materials and Methods

### Patient, Control, and Ethical Considerations

All experiments using human fetal membrane strictly followed the relevant regulations of the ethical experimental operation of Central South University. The study was reviewed, approved, and supervised by the Medical Ethics Committee of Central South University. The medical ethics committee unanimously voted (0:10) to approve this study. The data of a clinical auxiliary examination and imaging of one patient with pregnancy-related ADEM were collected. The healthy control was a 15-year-old pregnant student. She decided to have an abortion at 22 weeks of gestation, which was similar to the pregnancy term of the patient with pregnancy-related ADEM. The healthy control had no special medical history during the pregnancy period. The clinical characteristics of the healthy control matched those of the patient with ADEM. The patient and normal control signed informed consent, which was approved by the medical institutions.

### Case Presentation

**Figure 1:**
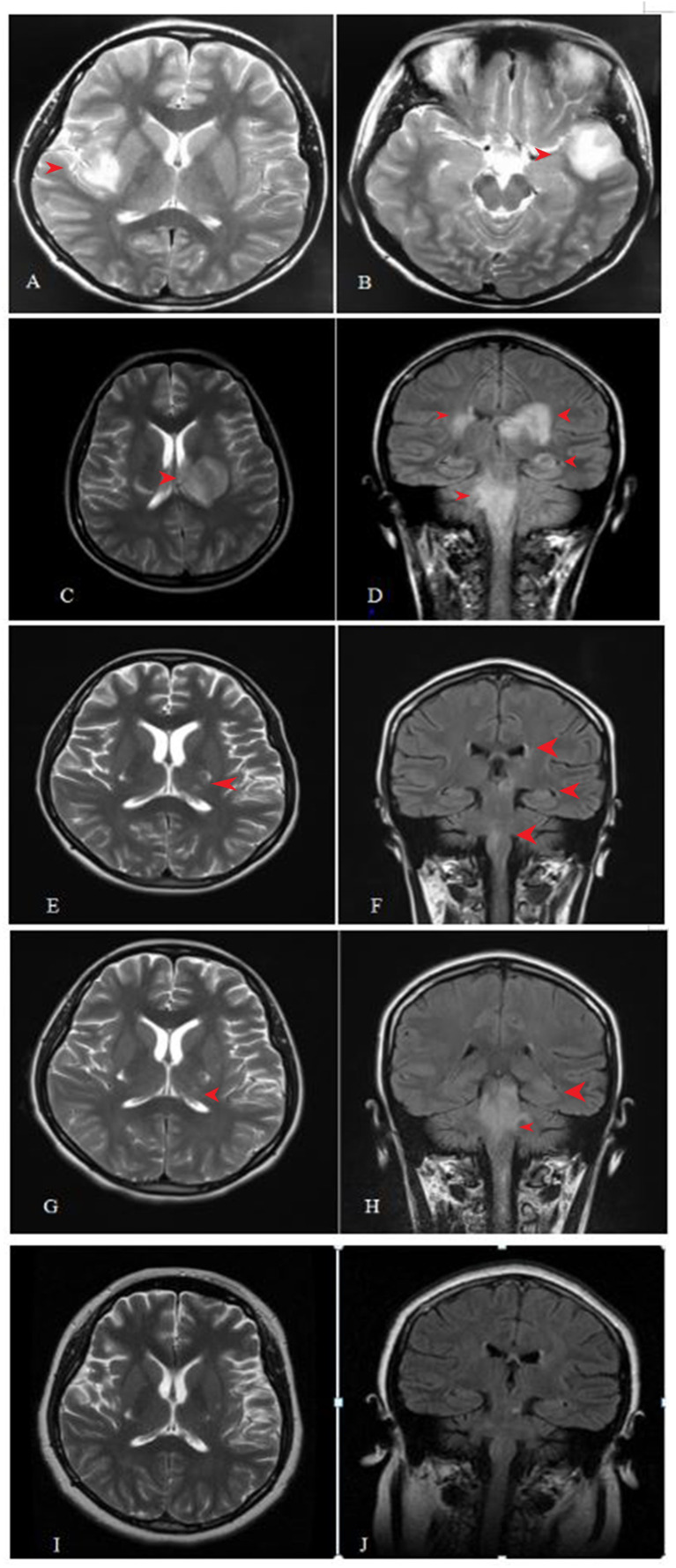
Cranial magnetic resonance imaging (MRI) examination during the process of disease. **(A, B)** lesions in left temporal lobe and right insular lob. **(C, D)** lesions in the medulla, pons, the right cerebral peduncle, bilateral posterior limb of internal capsule, bilateral Insula medialis, left temporal lobe, and right posterior horn of lateral ventricle. **(E, F)** lesion was significantly smaller than before in 7 days after abortion. **(G, H)** Some new demyelinating-like lesion was found in 26 days after abortion. **(I, J)** Lesions in brain stem, bilateral basal ganglia had disappeared which was observed by brain MRI scan at September 8, 2017 during following-up.

On August 8, 2016 (at 4 weeks of gestation, as reviewed retrospectively), an 18-year-old woman first presented with fever, headache, vomiting, and limb weakness; she experienced a cold before the onset of the disease. Serological tests for common infectious agents, including human T-cell lymphotropic viruses I and II; hepatitis A, B, and C viruses; human immunodeficiency viruses 1 and 2; herpes simplex viruses 1 and 2; Epstein–Barr virus; cytomegalovirus; rubella, measles, mumps, and poliomyelitis viruses; as well as *Mycoplasma pneumoniae*, *Chlamydia* spp., *Yersinia* spp., *Campylobacter* spp., *Rickettsia* spp., *Borrelia* spp., pathogens of toxoplasmosis and cryptococcosis, and *Coxiella burnetii*, were negative. Cerebrospinal fluid (CSF) examination showed a slightly elevated white blood cell count (**Table 1**). Brain magnetic resonance imaging (MRI) showed a lesion in the left temporal and right insular lobes (**Figures 1A, B**). The patient was diagnosed with viral meningoencephalitis. She recovered with regular acyclovir treatment and was discharged. On September 17, 2016 (at 9 weeks of gestation, as reviewed retrospectively), a subsequent relapse occurred, and the patient presented with headache, nausea, and vomiting. Thus, acyclovir was administered again, and the patient’s condition improved. However, on October 6, 2016, the patient lost consciousness and experienced respiratory failure. She was admitted to an intensive care unit, and based on MRI findings (**Figures 1C, D**) and CSF analysis (**Table 1**), a diagnosis of ADEM was established. The symptoms presented as alterations in the consciousness levels and behavior that could not be explained by fever, and brain MRI during the acute (3 months) phase indicated abnormalities consistent with demyelination, which supported the diagnosis of ADEM (33). In addition, tests for antibodies against proteins associated with demyelination (myelin oligodendrocyte glycoprotein, myelin basic protein, and AQP4), oligoclonal bands, autoimmune encephalitis, and paraneoplastic syndrome were negative in both the blood and CSF, which can distinguish ADEM from other demyelinating diseases to some extent. Surprisingly, an intrauterine pregnancy at 12 weeks of gestation was confirmed by abdominal ultrasound. After plasma exchange and gamma globulin (IVIG) therapy, the patient gradually recovered and was able to breathe spontaneously until November 11, 2020. A repeated brain MRI scan showed a significant reduction in the lesion size (**Figures 1E, F**). Thereafter, she decided to terminate the pregnancy at 20 weeks of gestation. The abortion occurred on December 1, 2016, and she experienced paroxysmal dizziness and rotation of visual objects for 10 days after the abortion. Multiple new demyelinating-like phenomena were observed by brain MRI (**Figures 1G, H**) on December 12, 2016, approximately 2 weeks after the abortion. She was again treated with methylprednisolone and IVIG. After a month of rehabilitation training, she was discharged with a better health condition. Lesions in brain stem, bilateral basal ganglia had disappeared which was observed by brain MRI scan at September 8, 2017 during following-up (**Figures 1I, J**). In the next 2 years, she gradually recovered, without recurrence. At present, she can complete simple daily activities, such as eating, combing her hair, and dressing. The detailed diagnosis and treatment processes are shown in **Table 2**.

**Table 1:** Cerebrospinal fluid findings.

Date	17/8	22/8	19/9	6/10	11/10	25/10	13/12		28/12
**CSF opening pressure(mmHg)**	230	NA	NA	150	280	85	150		150
**Cell count (cells/mm3)**	90	10	10	36	7	7	3		0
**Cell predominance**	NA	NA	NA	Monocytes	Monocytes	Monocytes	Monocytes		0
**CSF glucose(mmol/l)**	Normal	Normal	Normal	2.45	3.47	3.04	2.91		3.39
**CSF protein(mg/l)**	Normal	Normal	Normal	616.90	258.05	48.94	95.63		277.62

CSF, cerebrospinal fluid; NA, not available. Description according to report.

**Table 2:** The symptoms and treatment of patients at different courses in disease.

Date	Symptoms	Pregnancy state	Treatment
**August 8, 2016 to September 9, 2016**	Low-grade fever, severe headache, vomiting (August 8)	4-week gestation	Antiviral
**September 17, 2016 to September 29, 2016**	Hyperpyrexia, headache, nausea, vomiting (September 17)	9-week gestation	Antiviral
**October 10, 2016 to November 11, 2016**	Limb weakness (October 6)	12-week gestation	Acyclovir antiviral, cefotaxime
	Altered state of consciousness, epilepsy (October 9)		Suppressing epilepsy, IVIG, plasma exchange
	Respiratory failure (October 12)		Mechanical ventilation
**December 1, 2016 to February 9, 2017**	Paroxysmal dizziness and rotation of visual objects (December 10)	10 days after abortion	Methylprednisolone
	Headache, fever, limb weakness (December 25)	25 days after abortion	IVIG

### Flow Cytometry

During hospitalization (between December 1, 2016 and February 9, 2017), blood flow cytometry analysis was performed to observe the changes in circulating immune cell types after the abortion. Peripheral blood samples were taken on days 3, 5, 7, 14, 27, and 53 after the abortion, and flow cytometry was used to determine the distribution of the lymphocyte subsets present. Peripheral blood mononuclear cells (PBMCs) were separated by Ficoll-Paque density-gradient (34). The fresh separated PBMCs were used for flow cytometry analysis. Lymphocyte subpopulations were identified using combinations of the following antibodies: CD4(APC), CD3(FITC), CD8 (PE), CD28 (PerCP-Cy5.5), CD19(APC), CD56(APC), and CD45(PE-Cy7) (Beckton Dickinson San Jose, CA, USA). Photomultiplier tubes (PMTs) collected the fluorescent light activated by a solid-state laser of 488 nm (FITC, PE, PerCP), and 640 nm (APC) (35). After staining, samples were washed with phosphate buffer solution (PBS) and fixed with 2% paraformaldehyde. Staining was visualized using a CX31 microscope (OLYMPUS, Japan). Data were gathered in FACS Calibur flow cytometer (Becton Dickinson, San Jose, CA, USA) and analyzed with Flowjo_7.6 software (Tree Star, Inc., Ashland, OR). By detecting the expression of CD3, CD19, CD56, CD4, CD8, and CD28 extracellular markers in live cells, the lymphocyte percent was demonstrated with forward scatter (FSC)/side scatter (SSC) gating strategy.

### Western Blotting and Quantitative Real Time-PCR

Placenta and fetal membrane specimens were collected immediately after abortion and stored in liquid nitrogen following standard cryopreservation techniques (36). The expression of HMGB1, TLR4, Nf-κB, AQP4, IL-6, IL-4, TNF-α, and IL-2 at the protein and mRNA levels was detected in the samples of the fetal membrane (ADEM and normal) and placenta (ADEM and normal) by WB and Real-time qPCR, respectively.

Total proteins were extracted from the placenta and fetal membrane specimens using radioimmunoprecipitation assay lysis buffer (Sigma, St. Louis, MO, USA). Protein lysates (50 mg) were separated by sodium dodecyl sulfate polyacrylamide gel electrophoresis and then transferred to polyvinylidene difluoride membrane (Millipore, Bedford, MA, USA). The membrane was blocked with 5% skim milk in Tris-buffered saline with Tween 20 for 1 h. Further, the membrane was incubated with primary antibodies (all from Abcam, USA) against AQP4 (ab125049), HMGB1 (ab79823), TLR4 (ab13867), Nf-κB p65 (ab16502), IL-2 (ab9618), IL-4 (ab62351), IL-6 (ab233706), and TNF-α (ab215188), respectively, at 4°C overnight. The blots were incubated subsequently with Goat Anti-Rabbit IgG H&L (Alexa Fluor^®^ 488) (ab150077) for 2 h at room temperature. Reactive protein bands were detected using an enhanced chemiluminescence system (Santa Cruz Biotechnology). Band intensity was measured by ImageJ software (NIH, Bethesda, MD, USA) and protein expression levels were normalized to the matched total proteins or Actin (37).

To investigate gene expression, total RNA was extracted from the placenta and fetal membrane specimens using the Trizol reagent (Invitrogen, Carlsbad, CA, USA) following the manufacturer’s instructions. The cDNA was obtained using the reverse transcription kit (Thermo Fisher Scientific, Waltham, MA, United States) (38). The qPCR was performed using TaqMan Gene Expression Assay (Applied Biosystems). The specific primer sequences were presented in **Table 3**. qPCR was performed at the following cycling conditions: initial denaturation at 94°C for 10 min; denaturing at 94°C for 30 s, annealing at 53°C for 30 s, and extension at 72°C for 45 s, 40 cycles; final extension at 72°C for 5 min (39). The relative mRNA expression level was calculated using the 2^-△△^ Ct method.

**Table 3:** Reference genes for gene expression normalization.

Gene name	Forward primer sequence (5′-3′)	Reverse primer sequence (5′-3′)
***HMGB1***	5′-TTCTGCTCTGAGTATCGCCCAA-3′	5′-CCAGTTTCTTCGCAACATCACC-3′
***AQP4***	5′-CCCAGCAAACAAAAGGAAGCTAC-3′	5′-CTTCTTCTCCTCTCCCCGGTC-3′
***TLR4***	5′-AGTTGATCTACCAAGCCTTGAGT-3′	5′-GCTGGTTGTCCCAAAATCACTTT-3′
***NFKB1***	5′-AGGCTATCAGTCAGCGCATC-3′	5′-CACTGTCACCTGGAAGCAGA-3′
***IL-4***	5′-CACCTCCCAACTGCTTC-3′	5′-GTCTGTTACGGTCAACTCG-3′
***IL-6***	5′-AACAAATTCGGTACATCCTCGAC-3′	5′-ATTTTCACCAGGCAAGTCTCC-3′
***IL-2***	5′-AACTCCTGTCTTGCATTGCAC-3′	5′-GCTCCAGTTGTAGCTGTGTTT-3′
**TNF-α**	5′-AGCCCATGTTGTAGCAAACC-3′	5′-TGAGGTACAGGCCCTCTGAT-3′
***Actin***	5′-ACCCTGAAGTACCCCATCGAG-3′	5′- AGCACAGCCTGGATAGCAAC-3′

### Statistical Analysis

Three repeats were performed for each tissue sample. Data are expressed as the mean ± standard deviation (SD). All experimental data were analyzed using GraphPad Prism 5.0 (GraphPad Software, La Jolla, CA, USA). Since the biological sample size was one in this study, we present the data in a descriptive manner.

## Results

### Flow Cytometry

**Figure 2:**
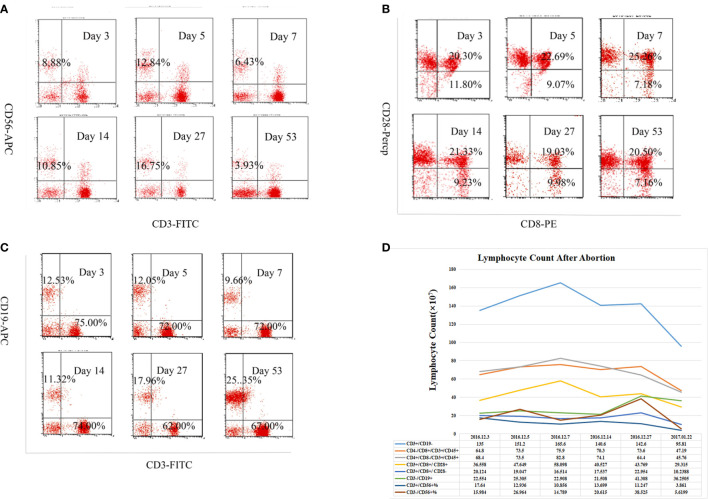
Alteration in subpopulation of peripheral lymphocyte in acute disseminated encephalomyelitis (ADEM) patient after abortion. The cells were stained for CD3, CD19, CD56, CD4, CD8, and CD28 with a viability dye. Relative frequencies (%) of **(A)** CD3-56+ NK cells, **(B)** CD8+CD28+ and CD8+CD28- T lymphocytes, **(C)** CD3+CD19- T lymphocytes, CD3-CD19+ B lymphocytes among all live lymphocytes were demonstrated with forward scatter (FSC)/side scatter (SSC) gating strategy on different times after abortion. **(D)** Absolute cell count of lymphoid subgroup (×10^7^) detect by flow cytometry in peripheral blood on different times after abortion. The absolute counts of CD3−CD19+ cells (total B lymphocytes) showed an upward trend, while the CD3+CD56+ natural killer T (NKT) cells showed a general downward trend.

Peripheral blood samples were collected from the patient with ADEM on days 3, 5, 7, 14, 27, and 53 after the abortion. The dynamic changes in lymphocyte subsets are shown in **Figures 2A–C**. On day 14 after the abortion, the absolute counts of CD3^−^CD19^+^ cells (total B lymphocytes) showed an upward trend. The absolute counts of CD3^+^CD56^+^ natural killer T (NKT) cells were low on days 3, 5, and 7 after the abortion but then increased on day 14 and gradually decreased thereafter, showing a general downward trend (**Figure 2D**).

### Western Blotting

**Figure 3:**
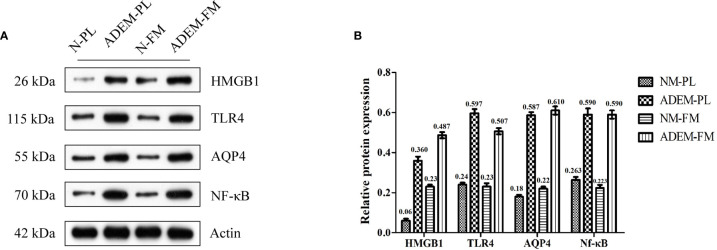
Protein expression levels of HMGB1/TLR4/Nf–κB/IL-6 pathway in fetal membrane and placenta. **(A)** The protein level of HMGB1, TLR4, AQP4, and Nf-kB in fetal membrane and placenta. Quantification of **(A)** is shown in **(B)**. Data are shown as mean ± SD from three repeats performed for each tissue sample and the mean values were added above the bar chart. N-FM, Normal-fetal membrane; ADEM-FM, ADEM-fetal membrane; N-PL, Normal- placenta; ADEM-PL, ADEM-placenta.

The potential roles of the HMGB1/TLR4/Nf-κB/IL-6 signaling pathway and AQP4 in the pathogenesis of pregnancy-related ADEM were explored by evaluating the relative expression levels of these proteins in the fetal membrane and placenta specimens. The results showed that the expression levels of HMGB1, TLR4, Nf-κB, and AQP4 were higher in the ADEM fetal membrane and placenta than in the normal fetal membrane and placenta (**Figure 3**).

**Figure 4:**
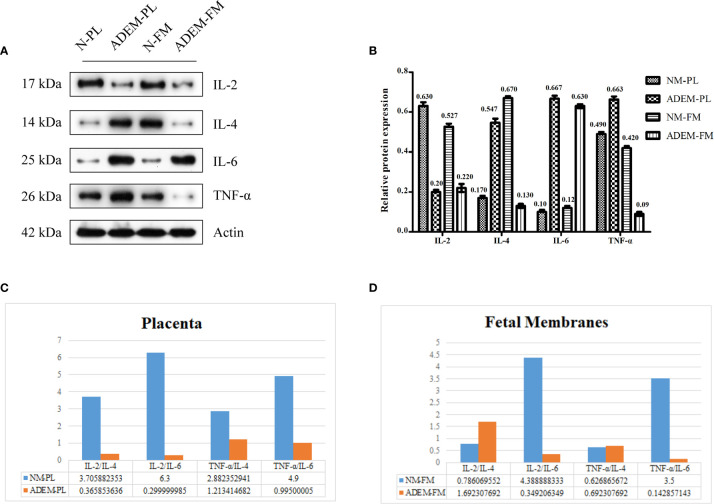
Protein expression levels of different cytokine in fetal membrane and placenta. **(A)** The protein level of IL-2, IL-4, IL-6, and TNF-α protein in fetal membrane and placenta. Quantification of **(A)** is shown in **(B)**. Data are shown as mean ± SD from three repeats performed for each tissue sample and the mean values were added above the bar chart. **(C, D)**, The calculated ratios of Th1/Th2 cytokines (IL-2/IL-4, TNF-α/IL-6, etc.). N-FM, Normal-fetal membrane; ADEM-FM, ADEM-fetal membrane; N-PL, Normal- placenta; ADEM-PL, ADEM-placenta.

Based on previous studies, cytokines related to the activation of Th1 (secreting TNF-α and IL-2) and Th2 cells (secreting IL-4 and IL-6) are increased in the CSF of ADEM patients (4, 5). Therefore, we chose TNF-α, I IL-2 to represent Th1 cytokines and IL-4, IL-6 to represent Th2 cytokines. The levels of the IL-6 protein were higher in the ADEM fetal membrane and placenta than in the normal fetal membrane and placenta. Conversely, the expression levels of the IL-2 protein were lower in the ADEM fetal membrane and placenta than in the normal fetal membrane and placenta (**Figures 4A, B** **)**. The levels of the TNF-α and IL-4 proteins were lower in the ADEM fetal membrane than in the normal fetal membrane and higher in the ADEM placenta than in the normal placenta. As Th1/Th2 cytokine ratios are more indicative of the Th1-to-Th2 shift than the absolute levels of each cytokine, we calculated the Th1/Th2 cytokine ratios (IL-2/IL-4, TNF-α/IL-6, etc.). ADEM placenta had lower Th1/Th2 ratios than those in the normal placenta, which is suggestive of a stronger bias toward type 2 cytokine dominance in placenta (**Figures 4C, D**). However, not all Th1/Th2 cytokines ratio in fetal membrane of ADEM patient decreased compared with those in the normal fetal membrane.

### Quantitative Real Time-PCR

**Figure 5:**
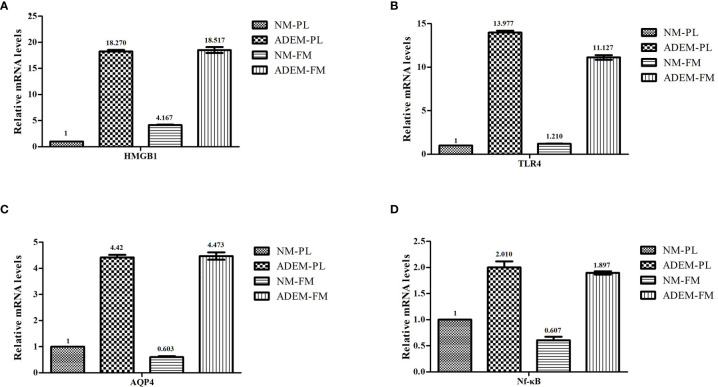
The mRNA expression levels in HMGB1/ TLR4/ Nf-KB/ IL-6 pathway in fetal membrane and placenta. The mRNA expression levels of HMGB1 **(A)**, TLR4 **(B)**, AQP4 **(C)**, and Nf-kB **(D)** in fetal membrane and placenta. Data are shown as mean ± SD from three repeats performed for each tissue sample and the mean values were added above the bar chart. N-FM, Normal-fetal membrane; ADEM-FM, ADEM-fetal membrane; N-PL, Normal- placenta; ADEM-PL, ADEM-placenta.

In addition to the relative protein expression levels of the HMGB1/TLR4/Nf-κB/IL-6 signaling pathway and AQP4, the relative mRNA expression levels were determined in the fetal membrane and placenta samples by qRT-PCR. Consistent with the results of WB, those of qPCR showed that the mRNA expression of *HMGB1*, *TLR4*, *NFKB1*, and *AQP4* was higher in the ADEM fetal membrane and placenta than in the normal fetal membrane and placenta (**Figure 5**).

**Figure 6:**
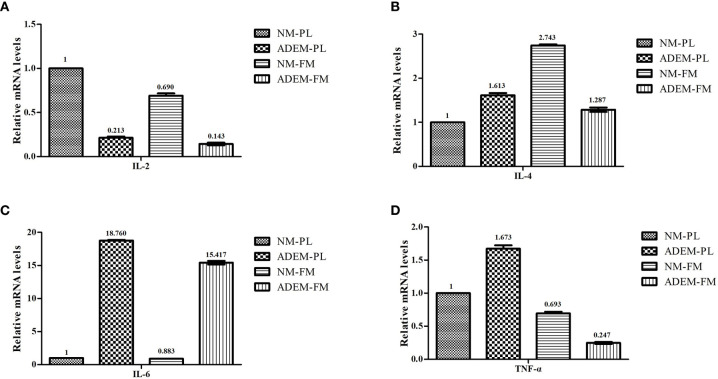
The mRNA expression levels of different cytokine in fetal membrane and placenta. The mRNA expression levels of IL-2 **(A)**, IL-4 **(B)**, IL-6 **(C)**, and TNF-α **(D)** in fetal membrane and placenta. Data are shown as mean ± SD from three repeats performed for each tissue sample and the mean values were added above the bar chart. N-FM, Normal-fetal membrane; ADEM-FM, ADEM-fetal membrane; N-PL, Normal- placenta; ADEM-PL, ADEM-placenta.

**Figure 6** shows that the *IL2* mRNA expression levels were lower in the ADEM fetal membrane and placenta than in the normal fetal membrane and placenta. The ADEM fetal membrane and placenta showed enhanced transcription of *IL6* compared with that in the normal fetal membrane and placenta. The expression of the *IL4* and *TNFA* mRNA was lower in the ADEM fetal membrane than in the normal fetal membrane and higher in the ADEM placenta than in the normal placenta.

## Discussion

### Relationship Between Pregnancy-Related Dynamic Immune Changes and Acute Disseminated Encephalomyelitis Pathogenesis

Demyelinating diseases of the CNS are autoimmune diseases and mainly include multiple sclerosis (MS), ADEM and NMOSD. In MS, the rate of relapse decreases during pregnancy and significantly rises during the first 3 months postpartum (18, 40), while NMOSD remains active during pregnancy, and the recurrence rates of fetal loss and preeclampsia increase over time (41, 42). In this case, the patient’s condition repeatedly worsened at 3 months gestion and after the abortion, which indicated that the onset of ADEM in this patient may be related to pregnancy. To date, the common feature of the previous reported pregnancy-related ADEM cases was that their clinical symptoms were exacerbated during pregnancy and improved by plasmapheresis (8–11, 43). Plasmapheresis relieves clinical symptoms by removing harmful circulating antibodies that may cause demyelination (8). In addition, during pregnancy, patients with ADEM show worsening symptoms, which are relieved after delivery or termination of pregnancy (10). Similar to previous reports, the patient with ADEM in our study experienced worsened conditions at 3 months of gestation, while plasmapheresis and IVIG therapy relieved clinical symptoms. However, mild symptoms recurred after an abortion at 5 months of gestation and were treated again with methylprednisolone and IVIG. Thus, the relationship between ADEM and pregnancy is complex and needs further study.

At present, it is believed that the pathogenesis of ADEM is related to the enhancement of humoral immunity, which is mediated by the increase in Th2 cytokines (4, 5, 9–11, 23, 25, 44). We measured the local cytokines in the placenta and fetal membrane from the patient with pregnancy-related ADEM and the healthy control after the abortion, and found decreased level of Th1/Th2 cytokine ratio in the placenta from ADEM patient compared with those in the control. Since maternal blood is in close contact with the fetal villous syncytiotrophoblasts after the establishment of the placental circulation, both systemic and local changes might be due to factors produced by the placenta and/or caused by direct contact of immune cells with placental trophoblast cells (15, 45). The local cytokines in the placenta may indirectly indicate a systemic Th2 shift. Th2 cytokines are related to B-cell proliferation, maturation, and antibody production (7). Their main functions are to mediate humoral immune responses, inhibit Th1 responses, and provide an immune protection to trophoblasts and the fetus, enabling pregnancy to successfully proceed (46). However, compared with the healthy control, not all Th1/Th2 cytokines ratio in fetal membrane of ADEM patients were decrease, which needs to be further investigated in larger sample size study.

Flow cytometry analysis revealed that the number of B cells increased after the abortion in this pregnancy-related ADEM patient, while the number of NKT cells decreased. The decrease of NK T cell may relate to pregnancy termination. Previous studies have shown that NKT cells are highly accumulated in the decidua in early pregnancy but decrease in the third trimester in both human and animal models (47, 48). In early pregnancy, NKT cells can be recruited to the implantation site during decidualization and stimulate trophoblasts to produce human chorionic gonadotropin and progesterone, thus leading T cells to differentiate into Th2 cells and regulate the Th1/Th2 balance (49, 50). However, since the diagnosis of our case was retrospective, we were unable to examine changes in lymphocyte subsets during the initial conception and the first manifestation of the disease.

### Role of the High Mobility Group B1/Toll-Like Receptor 4/Nf-κB/IL-6 Pathway and Aquaporin 4 in the Acute Disseminated Encephalomyelitis Fetal Membrane and Placenta

HMGB1, a non-histone DNA-binding protein, which is located in the nucleus, acts as a central molecule triggering and sustaining the cascading inflammatory response (51). As a late-stage inflammatory mediator, HMGB1 binds to a TLR, activating the immune system, and induces T-cell differentiation (52). Furthermore, activation of the HMGB1/TLR4/Nf-κB signaling pathway may promote the release of inflammatory cytokines, such as IL-6, TNF-α, and IL-1 (19). The HMGB1/TLR4/Nf-κB/IL-6 pathway is involved not only in viral responses but also in the pathogenesis of several inflammation-related diseases in the animal model, such as traumatic brain injury (TBI) (53) and lung injury induced by particulate matter (PM) 2.5 (54). In our study, we found increased level of HMGB1 in the embryonic membrane and placenta from our patient. Meanwhile, the downstream transcription factor Nf-κB and IL-6 were also remarkably elevated. These findings indicate that the immune response was abnormally activated at the maternal–fetal interface in the patient with ADEM. We assume that the locally elevated IL-6 in the placenta and fetal membrane of this patient may have been associated with elevated expression of IL-6 in the systemic circulation, and a further elevation of these abnormal immune inflammatory responses may be one of the pathogenic features of ADEM. IL-6, a pleiotropic factor, plays a dual role in inflammatory responses (55), acting as a promoter of inflammation, as well as a regulator of anti-inflammatory function (56). IL-6 promotes Th2-cell differentiation and simultaneously inhibits Th1-cell polarization *via* two independent molecular mechanisms that involve the nuclear factor of activated T cells (NFAT)-dependent and suppressor of cytokine signaling (SOCS)-1 pathways, respectively (57). Thus, we assume that increased IL-6 levels may also play a role in the regulation of immune inflammatory responses in ADEM. Unfortunately, blood samples were not collected at the time of abortion since the case was retrospective, and thus, our hypothesis needs to be further investigated.

Activation of the HMGB1/TLR4/Nf-κB/IL-6 pathway also regulates the expression of AQP4 (26). The increased expression of AQP4 was found in the placenta and fetal membrane from the patient in our study. The AQP4 expression pattern in a human placenta during gestation seems to suggest an important role for this protein in the regulation of the maternal–fetal fluid exchange between placental cells and fetal capillaries (31, 58). Moreover, an increase in AQP4 in the placenta and fetal membrane from the patient in our study, may be indirect evidence of the increase of AQP4 in the circulation and the brain (27, 59). Previous study has found a significant increase in the AQP4 protein in the whole brain during pregnancy in Sprague-Dawley rats (59–61), suggesting that the increase may be an adaptive adjustment during gestation. However, it has been revealed in rodent experiments that the upregulation of AQP4 in the brain increases the susceptibility of tissues to an autoimmune demyelinating disease attack during and after pregnancy (62). The expression of the AQP4 protein in the brain during pregnancy and postpartum does not cause edema under normal conditions but rather predisposes the brain to edema formation when a stressor disrupts the blood–brain barrier (BBB) (62). Accordingly, we assumed that the pregnancy-induced upregulation of the AQP4 protein in the CNS might aggravate brain edema and neurological defects upon BBB disruption during ADEM.

## Conclusion

In this study, we found that the local immune changes and the activation of the HMGB1/TLR4/Nf-κB/IL-6 pathway at the maternal–fetal interface in the ADEM patient compared to the healthy control, but the association between these changes and ADEM pathogenesis is not clear. Meanwhile, the relationship between an increased AQP4 expression and ADEM pathogenesis remains to be investigated. Because the case was reviewed retrospectively, there were limitations in our study, such as the lack of assessing T-cell polarization and intrathecal cytokines, as well as the lack of simultaneous evaluation of cytokine secretion into the blood of the patient during different periods of pregnancy. Further studies with a larger sample size are needed for comparison between healthy pregnancy and that in patients with ADEM. In addition, we recommend that specimens be properly preserved for subsequent immunohistochemistry and immunofluorescence studies.

## Data Availability Statement

The raw data supporting the conclusions of this article will be made available by the authors, without undue reservation.

## Ethics Statement

The studies involving human participants were reviewed and approved by the ethics committee of Second Xiangya Hospital. The patients/participants provided their written informed consent to participate in this study. Written informed consent was obtained from the individual(s) for the publication of any potentially identifiable images or data included in this article.

## Author Contributions

KQ performed the experiments. SD drafted the manuscript and analyzed the data. HZ and RT helped draft the manuscript. WL conceived and designed the study. All authors read and approved the final manuscript. All authors contributed to the article and approved the submitted version.

## Funding

This work was funded by the National Natural Science Foundation of China (#81571181).

## Conflict of Interest

The authors declare that the research was conducted in the absence of any commercial or financial relationships that could be construed as a potential conflict of interest.
